# 3,3,5,5-Tetra­methyl-3,5-disila-4,10-dioxatetra­cyclo­[5.5.1.0^2,6^.0^8,12^]tridecane-9,11-dione

**DOI:** 10.1107/S1600536809009222

**Published:** 2009-03-19

**Authors:** Peng-Peng Sheng, Jun-Ying Zhang, Lin Zhang

**Affiliations:** a Laboratory of Adhesives and in-situ Polymerization Technology, Beijing University of Chemical Technology, Beijing 100029, People’s Republic of China and Research Center of Laser Fusion, China Academy of Engineering Physics, Mianyang 621900, Sichuan, People’s Republic of China

## Abstract

The title compound, C_13_H_20_O_4_Si_2_, is a siloxane-functionalized norbornane anhydride. Both five-membered heterocyclic rings of the mol­ecule have a planar structure, whereas the two five-membered aliphatic rings assume envelope conformations. Weak inter­molecular C—H⋯O hydrogen bonding is present in the crystal structure.

## Related literature

For the synthesis and curing properties with the epoxy resin of silylnorbornane anhydrides, see: Eddy *et al.* (1990 ▶); Ryang (1983 ▶). For the preparation of the title complex by reacting 1,1,3,3-tetramethyldisiloxane and 5-norbornene-2,3-dicarb­ox­ylic acid anhydride in the presence of a platinum catalyst, see: Buese (1986 ▶); Eddy & Hallgren (1985 ▶); Ryang (1983 ▶); Swint & Buese (1991 ▶). In this reaction, the unsaturated anhydride was hydrosilylated with silicon hydride, see: Eddy & Hallgren (1987 ▶); Lewis & Uriarte (1990 ▶); Lewis (1990 ▶); Onopchenko & Sabourin (1987 ▶).
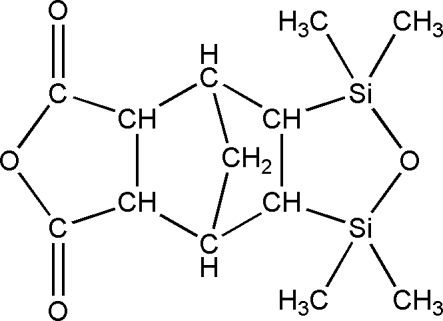


## Experimental

### 

#### Crystal data

C_13_H_20_O_4_Si_2_
*M* *_r_* = 296.47
Monoclinic, 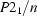

*a* = 8.0475 (16) Å
*b* = 12.047 (2) Å
*c* = 15.361 (3) Å
β = 95.84 (3)°
*V* = 1481.5 (5) Å^3^
*Z* = 4
Mo *K*α radiation
μ = 0.25 mm^−1^
*T* = 173 K
0.77 × 0.55 × 0.40 mm

#### Data collection

Rigaku R-AXIS RAPID IP area-detector diffractometer
Absorption correction: multi-scan (*ABSCOR*; Higashi, 1995 ▶) *T*_min_ = 0.833, *T*_max_ = 0.908
6539 measured reflections
3398 independent reflections
2921 reflections with *I* > 2σ(*I*)
*R*_int_ = 0.016

#### Refinement

*R*[*F* ^2^ > 2σ(*F* ^2^)] = 0.044
*wR*(*F* ^2^) = 0.102
*S* = 1.15
3398 reflections
172 parameters
H-atom parameters constrained
Δρ_max_ = 0.27 e Å^−3^
Δρ_min_ = −0.37 e Å^−3^

### 

Data collection: *RAPID-AUTO* (Rigaku, 2001 ▶); cell refinement: *RAPID-AUTO*; data reduction: *RAPID-AUTO*; program(s) used to solve structure: *SHELXS97* (Sheldrick, 2008 ▶); program(s) used to refine structure: *SHELXL97* (Sheldrick, 2008 ▶); molecular graphics: *XP* in *SHELXTL* (Sheldrick, 2008 ▶); software used to prepare material for publication: *SHELXL97*.

## Supplementary Material

Crystal structure: contains datablocks I, global. DOI: 10.1107/S1600536809009222/xu2490sup1.cif

Structure factors: contains datablocks I. DOI: 10.1107/S1600536809009222/xu2490Isup2.hkl

Additional supplementary materials:  crystallographic information; 3D view; checkCIF report

## Figures and Tables

**Table 1:** Hydrogen-bond geometry (Å, °)

*D*—H⋯*A*	*D*—H	H⋯*A*	*D*⋯*A*	*D*—H⋯*A*
C11—H11*A*⋯O3^i^	0.98	2.56	3.432 (2)	149
C12—H12*C*⋯O3^ii^	0.98	2.57	3.443 (2)	149

Symmetry codes: (i) 
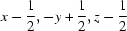
; (ii) 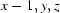.

**Fig. 1.**
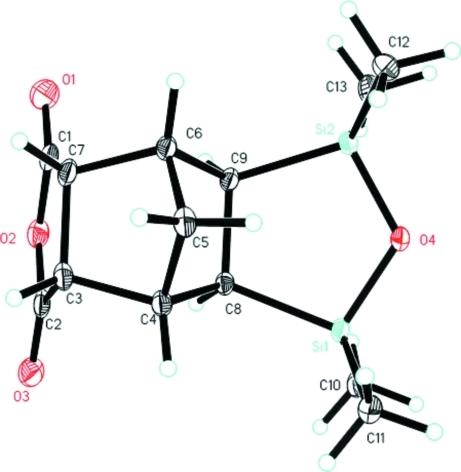
The molecular structure of the title compound at 50% probability level.

## supplementary crystallographic information

### Comment

Recently, we are interested in the synthesis and curing properties with epoxy resin of silylnorbornane anhydrides. The cured products show improved thermal and physical properties as compared to conventional curing agents (Eddy *et al.*, 1990; Ryang, 1983). The title complex was provided by reacting 1,1,3,3-tetramethyldisiloxane and 5-norbornene-2,3-dicarboxylic acid anhydride in the presence of a platinum catalyst (Buese, 1986; Eddy & Hallgren, 1985; Ryang, 1983; Swint & Buese,1991). In this reaction, the unsaturated anhydride was hydrosilylated with silicon hydride (Eddy & Hallgren, 1987; Lewis & Uriarte, 1990; Lewis, 1990; Onopchenko & Sabourin, 1987).

In the title compound, the two Si atoms in tetramethyldisiloxane are linked into a ring by carbon-silicon linkages by two C atoms (Fig. 1). Both five-membered heterocyclic rings of the molecule have planar structure, whereas two five-membered aliphatic rings assume the envelope conformation. The weak intermolecular C—H···O hydrogen bonding presents in the crystal structure (Table 1).

### Experimental

Synthetic reaction was performed in refluxing toluene under hermetic condition. Toluene was dried over appropriate drying agent and distilled prior to use. There was added 10 drops platinum catalyst to a mixture while it was being stirred of 36.08 g (0.22 mole) of 5-norbornene-2,3- dicarboxylic acid anhydride, 13.4 g (0.1 mole) of 1,1,3,3-tetramethyldisiloxane and 150 ml of toluene. The resulting mixture was heated to 70°C for 8 h and then 100°C overnight. After cooling, filtration, removal of the solvent under vacuum and addition of dry diethyl ether resulted in the precipitation of white powder. Colourless crystals of the title compound suitable for X-ray structure analysis were obtained by crystallization in appropriate solvent.

### Refinement

All H atoms were fixed geometrically and treated as riding atoms with distances C—H = 0.98 Å (CH_3_), 0.99 Å (CH_2_) or 1.000 Å (CH) with *U*_iso_(H) = 1.2U_eq_(C) or 1.5U_eq_(C).

### Crystal data

**Table:**

C_13_H_20_O_4_Si_2_	*F*(000) = 632
*M**_r_* = 296.47	*D*_x_ = 1.329 Mg m^−^^3^
Monoclinic, *P*2_1_/*n*	Mo *K*α radiation, λ = 0.71073 Å
Hall symbol: -P 2yn	Cell parameters from 687 reflections
*a* = 8.0475 (16) Å	θ = 2.2–27.5°
*b* = 12.047 (2) Å	µ = 0.25 mm^−^^1^
*c* = 15.361 (3) Å	*T* = 173 K
β = 95.84 (3)°	Block, colourless
*V* = 1481.5 (5) Å^3^	0.77 × 0.55 × 0.40 mm
*Z* = 4	

### Data collection

**Table:**

Rigaku R-AXIS RAPID IP area-detector diffractometer	3398 independent reflections
Radiation source: rotating anode	2921 reflections with *I* > 2σ(*I*)
graphite	*R*_int_ = 0.016
ω scans at fixed χ = 45°	θ_max_ = 27.5°, θ_min_ = 2.2°
Absorption correction: multi-scan (*ABSCOR*; Higashi, 1995)	*h* = −10→10
*T*_min_ = 0.833, *T*_max_ = 0.908	*k* = −15→15
6539 measured reflections	*l* = −19→19

### Refinement

**Table:**

Refinement on *F*^2^	Primary atom site location: structure-invariant direct methods
Least-squares matrix: full	Secondary atom site location: difference Fourier map
*R*[*F*^2^ > 2σ(*F*^2^)] = 0.044	Hydrogen site location: inferred from neighbouring sites
*wR*(*F*^2^) = 0.102	H-atom parameters constrained
*S* = 1.15	*w* = 1/[σ^2^(*F*_o_^2^) + (0.0539*P*)^2^] where *P* = (*F*_o_^2^ + 2*F*_c_^2^)/3
3398 reflections	(Δ/σ)_max_ < 0.001
172 parameters	Δρ_max_ = 0.27 e Å^−^^3^
0 restraints	Δρ_min_ = −0.37 e Å^−^^3^

### Special details

**Table:**

Geometry. All e.s.d.'s (except the e.s.d. in the dihedral angle between two l.s. planes) are estimated using the full covariance matrix. The cell e.s.d.'s are taken into account individually in the estimation of e.s.d.'s in distances, angles and torsion angles; correlations between e.s.d.'s in cell parameters are only used when they are defined by crystal symmetry. An approximate (isotropic) treatment of cell e.s.d.'s is used for estimating e.s.d.'s involving l.s. planes.
Refinement. Refinement of *F*^2^ against ALL reflections. The weighted *R*-factor *wR* and goodness of fit *S* are based on *F*^2^, conventional *R*-factors *R* are based on *F*, with *F* set to zero for negative *F*^2^. The threshold expression of *F*^2^ > σ(*F*^2^) is used only for calculating *R*-factors(gt) *etc*. and is not relevant to the choice of reflections for refinement. *R*-factors based on *F*^2^ are statistically about twice as large as those based on *F*, and *R*- factors based on ALL data will be even larger.

### Fractional atomic coordinates and isotropic or equivalent isotropic displacement parameters (Å^2^)

**Table:**

	*x*	*y*	*z*	*U*_iso_*/*U*_eq_	
Si1	0.16741 (6)	0.26208 (4)	0.90062 (3)	0.02159 (13)	
Si2	−0.13909 (6)	0.22004 (4)	0.97218 (3)	0.01986 (13)	
O1	0.1060 (2)	0.08686 (13)	1.26700 (10)	0.0447 (4)	
O2	0.34875 (17)	0.10991 (11)	1.21113 (8)	0.0295 (3)	
O3	0.57506 (18)	0.17797 (12)	1.15730 (9)	0.0371 (4)	
O4	−0.03918 (16)	0.25012 (11)	0.88621 (8)	0.0268 (3)	
C1	0.1966 (3)	0.14869 (17)	1.23476 (11)	0.0295 (4)	
C2	0.4400 (2)	0.19586 (15)	1.17782 (11)	0.0264 (4)	
C3	0.3389 (2)	0.30054 (14)	1.17335 (11)	0.0239 (4)	
H3A	0.3993	0.3615	1.2076	0.029*	
C4	0.2776 (2)	0.33916 (14)	1.07905 (11)	0.0216 (4)	
H4A	0.3590	0.3866	1.0509	0.026*	
C5	0.1164 (2)	0.39877 (14)	1.09749 (11)	0.0239 (4)	
H5A	0.0507	0.4253	1.0435	0.029*	
H5B	0.1368	0.4605	1.1397	0.029*	
C6	0.0379 (2)	0.29693 (14)	1.13743 (11)	0.0222 (4)	
H6A	−0.0747	0.3105	1.1575	0.027*	
C7	0.1770 (2)	0.26977 (15)	1.21202 (11)	0.0254 (4)	
H7A	0.1630	0.3151	1.2653	0.031*	
C8	0.2102 (2)	0.23867 (14)	1.02310 (10)	0.0196 (3)	
H8A	0.2885	0.1746	1.0341	0.024*	
C9	0.0397 (2)	0.21144 (13)	1.06256 (10)	0.0191 (3)	
H9A	0.0451	0.1348	1.0876	0.023*	
C10	0.2621 (3)	0.15072 (18)	0.83835 (13)	0.0359 (5)	
H10A	0.2381	0.1647	0.7755	0.054*	
H10B	0.2149	0.0788	0.8528	0.054*	
H10C	0.3832	0.1498	0.8539	0.054*	
C11	0.2318 (2)	0.40101 (16)	0.86415 (12)	0.0299 (4)	
H11A	0.2057	0.4074	0.8006	0.045*	
H11B	0.3523	0.4105	0.8793	0.045*	
H11C	0.1716	0.4585	0.8933	0.045*	
C12	−0.2961 (2)	0.32903 (15)	0.98616 (12)	0.0278 (4)	
H12A	−0.3828	0.3271	0.9365	0.042*	
H12B	−0.2418	0.4019	0.9888	0.042*	
H12C	−0.3470	0.3158	1.0405	0.042*	
C13	−0.2450 (3)	0.08331 (15)	0.95812 (14)	0.0348 (5)	
H13A	−0.3371	0.0884	0.9114	0.052*	
H13B	−0.2890	0.0621	1.0129	0.052*	
H13C	−0.1647	0.0273	0.9427	0.052*	

### Atomic displacement parameters (Å^2^)

**Table:**

	*U*^11^	*U*^22^	*U*^33^	*U*^12^	*U*^13^	*U*^23^
Si1	0.0194 (2)	0.0322 (3)	0.0130 (2)	0.0056 (2)	0.00060 (17)	−0.00032 (18)
Si2	0.0180 (2)	0.0239 (2)	0.0170 (2)	0.00166 (18)	−0.00122 (17)	−0.00031 (17)
O1	0.0419 (9)	0.0574 (10)	0.0347 (8)	−0.0030 (7)	0.0025 (7)	0.0191 (7)
O2	0.0328 (8)	0.0327 (7)	0.0219 (6)	0.0063 (6)	−0.0031 (5)	0.0023 (5)
O3	0.0284 (8)	0.0543 (9)	0.0280 (7)	0.0140 (7)	0.0002 (6)	0.0065 (6)
O4	0.0209 (7)	0.0447 (8)	0.0142 (6)	0.0031 (6)	−0.0012 (5)	0.0008 (5)
C1	0.0314 (10)	0.0421 (11)	0.0141 (8)	0.0028 (8)	−0.0025 (7)	0.0031 (7)
C2	0.0281 (10)	0.0355 (10)	0.0139 (8)	0.0037 (8)	−0.0066 (7)	0.0004 (7)
C3	0.0238 (9)	0.0289 (9)	0.0175 (8)	0.0026 (7)	−0.0046 (7)	−0.0023 (7)
C4	0.0210 (9)	0.0251 (8)	0.0177 (8)	0.0024 (7)	−0.0022 (7)	0.0000 (6)
C5	0.0257 (9)	0.0243 (9)	0.0206 (8)	0.0051 (7)	−0.0033 (7)	−0.0031 (6)
C6	0.0212 (9)	0.0300 (9)	0.0150 (8)	0.0063 (7)	0.0004 (6)	−0.0022 (6)
C7	0.0278 (10)	0.0351 (10)	0.0128 (8)	0.0045 (8)	−0.0009 (7)	−0.0035 (7)
C8	0.0180 (8)	0.0258 (8)	0.0146 (7)	0.0066 (7)	−0.0004 (6)	−0.0011 (6)
C9	0.0212 (8)	0.0220 (8)	0.0139 (8)	0.0042 (6)	0.0005 (6)	−0.0009 (6)
C10	0.0392 (12)	0.0486 (12)	0.0206 (9)	0.0149 (10)	0.0056 (8)	−0.0044 (8)
C11	0.0270 (10)	0.0403 (11)	0.0218 (9)	0.0021 (8)	0.0003 (7)	0.0042 (7)
C12	0.0215 (9)	0.0322 (9)	0.0294 (10)	0.0058 (7)	0.0018 (7)	0.0022 (7)
C13	0.0357 (11)	0.0292 (10)	0.0373 (11)	−0.0018 (8)	−0.0078 (9)	−0.0021 (8)

### Geometric parameters (Å, °)

**Table:**

Si1—O4	1.6609 (14)	C5—H5A	0.9900
Si1—C11	1.856 (2)	C5—H5B	0.9900
Si1—C10	1.856 (2)	C6—C9	1.545 (2)
Si1—C8	1.8988 (17)	C6—C7	1.553 (2)
Si2—O4	1.6547 (14)	C6—H6A	1.0000
Si2—C12	1.8501 (18)	C7—H7A	1.0000
Si2—C13	1.8570 (19)	C8—C9	1.589 (2)
Si2—C9	1.8977 (18)	C8—H8A	1.0000
O1—C1	1.185 (2)	C9—H9A	1.0000
O2—C1	1.393 (2)	C10—H10A	0.9800
O2—C2	1.396 (2)	C10—H10B	0.9800
O3—C2	1.182 (2)	C10—H10C	0.9800
C1—C7	1.505 (3)	C11—H11A	0.9800
C2—C3	1.499 (3)	C11—H11B	0.9800
C3—C7	1.531 (3)	C11—H11C	0.9800
C3—C4	1.553 (2)	C12—H12A	0.9800
C3—H3A	1.0000	C12—H12B	0.9800
C4—C5	1.535 (2)	C12—H12C	0.9800
C4—C8	1.551 (2)	C13—H13A	0.9800
C4—H4A	1.0000	C13—H13B	0.9800
C5—C6	1.536 (2)	C13—H13C	0.9800
			
O4—Si1—C11	110.16 (8)	C7—C6—H6A	114.5
O4—Si1—C10	109.06 (9)	C1—C7—C3	104.57 (15)
C11—Si1—C10	110.76 (10)	C1—C7—C6	115.23 (15)
O4—Si1—C8	101.37 (8)	C3—C7—C6	103.91 (14)
C11—Si1—C8	113.90 (8)	C1—C7—H7A	110.9
C10—Si1—C8	111.15 (8)	C3—C7—H7A	110.9
O4—Si2—C12	109.29 (8)	C6—C7—H7A	110.9
O4—Si2—C13	110.85 (9)	C4—C8—C9	102.53 (13)
C12—Si2—C13	109.41 (10)	C4—C8—Si1	116.81 (12)
O4—Si2—C9	101.65 (7)	C9—C8—Si1	109.40 (11)
C12—Si2—C9	115.46 (8)	C4—C8—H8A	109.3
C13—Si2—C9	109.96 (8)	C9—C8—H8A	109.3
C1—O2—C2	110.85 (15)	Si1—C8—H8A	109.3
Si2—O4—Si1	118.28 (8)	C6—C9—C8	102.71 (13)
O1—C1—O2	119.50 (18)	C6—C9—Si2	116.39 (12)
O1—C1—C7	130.70 (19)	C8—C9—Si2	109.22 (11)
O2—C1—C7	109.81 (16)	C6—C9—H9A	109.4
O3—C2—O2	119.65 (17)	C8—C9—H9A	109.4
O3—C2—C3	130.63 (19)	Si2—C9—H9A	109.4
O2—C2—C3	109.71 (16)	Si1—C10—H10A	109.5
C2—C3—C7	104.96 (15)	Si1—C10—H10B	109.5
C2—C3—C4	114.45 (14)	H10A—C10—H10B	109.5
C7—C3—C4	103.46 (14)	Si1—C10—H10C	109.5
C2—C3—H3A	111.2	H10A—C10—H10C	109.5
C7—C3—H3A	111.2	H10B—C10—H10C	109.5
C4—C3—H3A	111.2	Si1—C11—H11A	109.5
C5—C4—C8	102.28 (14)	Si1—C11—H11B	109.5
C5—C4—C3	99.31 (14)	H11A—C11—H11B	109.5
C8—C4—C3	110.06 (14)	Si1—C11—H11C	109.5
C5—C4—H4A	114.5	H11A—C11—H11C	109.5
C8—C4—H4A	114.5	H11B—C11—H11C	109.5
C3—C4—H4A	114.5	Si2—C12—H12A	109.5
C4—C5—C6	95.15 (13)	Si2—C12—H12B	109.5
C4—C5—H5A	112.7	H12A—C12—H12B	109.5
C6—C5—H5A	112.7	Si2—C12—H12C	109.5
C4—C5—H5B	112.7	H12A—C12—H12C	109.5
C6—C5—H5B	112.7	H12B—C12—H12C	109.5
H5A—C5—H5B	110.2	Si2—C13—H13A	109.5
C5—C6—C9	101.53 (13)	Si2—C13—H13B	109.5
C5—C6—C7	99.69 (14)	H13A—C13—H13B	109.5
C9—C6—C7	110.38 (14)	Si2—C13—H13C	109.5
C5—C6—H6A	114.5	H13A—C13—H13C	109.5
C9—C6—H6A	114.5	H13B—C13—H13C	109.5
			
C12—Si2—O4—Si1	123.01 (10)	C4—C3—C7—C6	−1.66 (17)
C13—Si2—O4—Si1	−116.32 (10)	C5—C6—C7—C1	−148.19 (15)
C9—Si2—O4—Si1	0.51 (10)	C9—C6—C7—C1	−41.9 (2)
C11—Si1—O4—Si2	−122.87 (10)	C5—C6—C7—C3	−34.41 (16)
C10—Si1—O4—Si2	115.37 (10)	C9—C6—C7—C3	71.83 (17)
C8—Si1—O4—Si2	−1.94 (10)	C5—C4—C8—C9	−33.12 (15)
C2—O2—C1—O1	−177.51 (17)	C3—C4—C8—C9	71.70 (16)
C2—O2—C1—C7	2.98 (19)	C5—C4—C8—Si1	86.45 (15)
C1—O2—C2—O3	177.48 (16)	C3—C4—C8—Si1	−168.72 (12)
C1—O2—C2—C3	−3.49 (19)	O4—Si1—C8—C4	−113.01 (13)
O3—C2—C3—C7	−178.57 (19)	C11—Si1—C8—C4	5.26 (15)
O2—C2—C3—C7	2.54 (18)	C10—Si1—C8—C4	131.21 (14)
O3—C2—C3—C4	68.7 (3)	O4—Si1—C8—C9	2.82 (12)
O2—C2—C3—C4	−110.17 (17)	C11—Si1—C8—C9	121.09 (12)
C2—C3—C4—C5	150.76 (16)	C10—Si1—C8—C9	−112.96 (12)
C7—C3—C4—C5	37.17 (16)	C5—C6—C9—C8	36.63 (15)
C2—C3—C4—C8	43.9 (2)	C7—C6—C9—C8	−68.39 (17)
C7—C3—C4—C8	−69.65 (17)	C5—C6—C9—Si2	−82.62 (15)
C8—C4—C5—C6	55.13 (15)	C7—C6—C9—Si2	172.36 (12)
C3—C4—C5—C6	−57.91 (15)	C4—C8—C9—C6	−2.16 (15)
C4—C5—C6—C9	−56.45 (15)	Si1—C8—C9—C6	−126.78 (11)
C4—C5—C6—C7	56.84 (14)	C4—C8—C9—Si2	121.97 (11)
O1—C1—C7—C3	179.3 (2)	Si1—C8—C9—Si2	−2.65 (13)
O2—C1—C7—C3	−1.26 (18)	O4—Si2—C9—C6	117.15 (13)
O1—C1—C7—C6	−67.3 (3)	C12—Si2—C9—C6	−1.00 (16)
O2—C1—C7—C6	112.13 (17)	C13—Si2—C9—C6	−125.37 (14)
C2—C3—C7—C1	−0.75 (17)	O4—Si2—C9—C8	1.49 (12)
C4—C3—C7—C1	119.55 (15)	C12—Si2—C9—C8	−116.66 (12)
C2—C3—C7—C6	−121.95 (14)	C13—Si2—C9—C8	118.97 (12)

### Hydrogen-bond geometry (Å, °)

**Table:**

*D*—H···*A*	*D*—H	H···*A*	*D*···*A*	*D*—H···*A*
C11—H11A···O3^i^	0.98	2.56	3.432 (2)	149
C12—H12C···O3^ii^	0.98	2.57	3.443 (2)	149

Symmetry codes: (i) *x*−1/2, −*y*+1/2, *z*−1/2; (ii) *x*−1, *y*, *z*.
